# “Love me, parents!”: impact evaluation of a national social and behavioral change communication campaign on maternal health outcomes in Tanzania

**DOI:** 10.1186/s12884-017-1470-x

**Published:** 2017-09-15

**Authors:** Michelle R. Kaufman, Jennifer J. Harman, Marina Smelyanskaya, Jennifer Orkis, Robert Ainslie

**Affiliations:** 1 Johns Hopkins Bloomberg School of Public Health, Department of Health, Behavior & Society, 624 N. Broadway, Baltimore, MD 21205 USA; 2 Colorado State University, Department of Psychology, 219 Behavioral Sciences Building, Fort Collins, CO 80523-1876 USA; 3 Johns Hopkins Center for Communication Programs, 111 Market Place, Suite 310, Baltimore, MD 21202 USA

**Keywords:** Maternal health, Social and behavioral change communication, Tanzania, Birth planning, Antenatal care, Low-income setting, Women’s health

## Abstract

**Background:**

Despite marked improvements over the last few decades, maternal mortality in Tanzania remains among the world’s highest at 454 maternal deaths per 100,000 live births. Many factors contribute to this disparity, such as a lack of attendance at antenatal care (ANC) services and low rates of delivery at a health facility with a skilled provider. The *Wazazi Nipendeni* (Love me, parents) social and behavioral change communication campaign was launched in Tanzania in 2012 to improve a range of maternal health outcomes, including individual birth planning, timely ANC attendance, and giving birth in a healthcare facility.

**Methods:**

An evaluation to determine the impact of the national *Wazazi Nipendeni* campaign was conducted in five purposively selected regions of Tanzania using exit interviews with pregnant and post-natal women attending ANC clinics. A total of 1708 women were interviewed regarding campaign exposure, ANC attendance, and individual birth planning.

**Results:**

Over one third of interviewed women (35.1%) reported exposure to the campaign in the last month. The more sources from which women reported hearing the *Wazazi Nipendeni* message, the more they planned for the birth of their child (β = 0.08, *p* = .001). Greater numbers of types of exposure to the *Wazazi Nipendeni* message was associated with an increase in ANC visits (β = 0.05, *p =* .004). Intervention exposure did not significantly predict the timing of the first ANC visit or HIV testing in the adjusted model, however, findings showed that exposure did predict whether women delivered at a health care facility (or not) and whether they tested for HIV with a partner in the unadjusted models.

**Conclusions:**

The *Wazazi Nipendeni* campaign shows promise that such a behavior change communication intervention could lead to better pregnancy and childbirth outcomes for women in low resource settings. For outcomes such as HIV testing, message exposure showed some promising effects, but demographic variables such as age and socioeconomic status appear to be important as well.

## Background

While significant progress has been achieved in reducing maternal and child mortality in sub-Saharan Africa, not all countries in the region reached the Millennium Development Goals (MDGs) by the 2015 deadline [1]. MDG targets proposed a global reduction in the maternal mortality ratio by three fourths and a decrease in childhood mortality to 31 deaths per 1000 by 2015 [2]. On both targets, sub-Saharan Africa continues to remain significantly behind, with maternal mortality twice as high as the proposed goal of three quarters reduction in incidence and the early childhood mortality indicator at 178 deaths per 1000 [2]. The World Health Organization (WHO) recommends a number of strategies and best practices to address high maternal and child mortality, including early (within the first trimester) and consistent attendance (at least 4 visits) at antenatal care (ANC) services and delivery in a health facility [3].

In 2009, the Campaign on Accelerated Reduction of Maternal Mortality in Africa (CARMMA) was initiated by the African Union Commission [4]. Since then, CARMMA has been launched in 37 Union member states (in June 2011 in Tanzania) and seeks to improve both maternal and infant mortality indicators. Following these regional developments and in anticipation of the MDG deadline, UNICEF, in partnership with USAID, launched a call to action in June 2012 for “a promise renewed” to address child survival and development [2, 5]. While many maternal mortality programs are in existence, there is a lack of evaluation of such programs of how they actually impact health behaviors during pregnancy and reduce maternal and neonatal mortality [6]. The call to action therefore emphasized increased monitoring and evaluation of such programs.

### Maternal and child health in Tanzania

At 454 maternal deaths per 100,000 live births, Tanzania’s maternal mortality ratio remains one of the world’s highest [7]. Mortality rates for children under 5 years old also remain high. Despite efforts to reduce these indicators by two thirds since 1990, infant deaths in the country remain at 51 per 1000 live births; neonatal deaths are at 26 per 1000; and post - neonatal mortality is 25 per 1000 [7, 8].

Infectious disease continues to contribute to these disparities in Tanzania. HIV prevalence among pregnant women is 6.8%, compared to 5.1% prevalence among the general population, and only about 70% of pregnant women are reportedly reached with prevention of mother to child transmission (PMTCT) interventions [9, 10]. Furthermore, only 15% of pregnant women attend ANC in their first trimester; approximately 43% of women achieve the recommended minimum of four ANC visits; and only half of births take place at health facilities in the presence of a trained healthcare provider [7]. A recent study conducted in an urban Tanzanian hospital confirms that delays in seeking care and a complete lack of ANC attendance were responsible for up to 84% of maternal deaths [11].

### Behavior change communication to reduce maternal mortality

Exposure of mothers and the community at large to information about nutrition during pregnancy, birth preparedness, and care and treatment modalities during antenatal visits has been shown to increase skilled birth attendance and malaria prophylaxis uptake during pregnancy, thus resulting in positive outcomes for both mother and baby [12, 13]. In addition, birth preparedness messaging and awareness of danger signs during pregnancy have been stressed as effective strategies for maternal mortality reduction [14]. Further, knowledge of complications during pregnancy has been significantly associated with birth preparedness in other studies [15]. Evaluations of well-designed mass media campaigns have consistently found a positive influence of communication on behavioral change, including maternal health behaviors, with campaigns utilizing multiple, mutually reinforcing channels of communication yielding the best results [12, 13, 16–19].

### The Wazazi Nipendeni Campaign

*Wazazi Nipendeni,* or “Love me, parents”*—*a national social and behavioral change communication campaign*—*was launched in Tanzania to answer the local and global calls for improving maternal and early childhood outcomes. The campaign utilized an integrated, multi-channel strategic communication approach to reach pregnant women and their partners with the goal of influencing knowledge, skills, and behaviors associated with improved maternal and child health outcomes. The campaign integrated a spectrum of health behaviors to provide a comprehensive approach to promotion of healthy pregnancy and safe delivery, including antenatal care, and HIV and malaria prevention and treatment. The specific messages of the campaign encouraged 1) early and complete ANC attendance; 2) pregnant woman and partner testing for HIV, with subsequent enrollment in PMTCT services if positive; 3) receiving two doses of sulfadoxine/pyrimethamine (SP) to prevent malaria in pregnancy; 4) nightly use of insecticide-treated mosquito nets (ITNs); 5) creation of an individual birth plan (IBP); and 6) delivery at a health facility with a skilled provider.

The campaign’s design was informed by Social Cognitive Theory [20], which emphasizes the importance of reciprocal determinism: while environmental factors influence groups and individuals, individuals and groups can also influence their environments and regulate their own behavior. The theory considers both a person’s individual capacity to interact with their environment, as well as the capacity for collective action to bring about a desired outcome within a particular group. A key component of the theory is observational learning, in which one learns to perform new behaviors by exposure to interpersonal or media portrayals of the behaviors, especially through modeling by peers whom the observers feel are similar to themselves. Attention, retention, production, and motivation are important processes within observational learning.

Key behaviors targeted by the *Wazazi Nipendeni* campaign were depicted in scenarios in which characters representative of the target audience – and of their larger communities – engaged in the health behaviors of interest, thereby increasing self and collective efficacy to perform these actions. The scenarios were broadcast via radio, television, and print media. In one radio spot, for example, a pregnant woman tells her mother-in-law about her pregnancy, who in turn reacts with unbridled joy and enthusiasm. The mother-in-law encourages her daughter-in-law to tell her husband and go for her first ANC visit, thereby modeling early disclosure of pregnancy, family support, the importance of male involvement, and the need for early ANC attendance. Another radio spot portrays a pregnant woman convincing her partner to test for HIV. After a period of comical yet realistic reluctance, her husband agrees. By modeling the conversation itself, the spot aims to build the skills and self-efficacy needed to initiate these types of conversations. The name of the campaign itself also implies collective action; the literal translation of “wazazi” is “parents.” The term is also conceived as societal, with the whole community playing a part in healthy pregnancies and deliveries.

The campaign launched in November 2012, with messages communicated through radio and TV spots, billboards, magazine articles and advertisements, and a variety of health facility and promotional materials. TV spots focused on malaria prevention in pregnancy, IBP, and PMTCT were aired on six stations with a frequency of three television spots per station per day between November 2012 and March 2013 and again between July and December 2013. A popular reality TV show featuring grandmothers – an influential group in Tanzanian society – incorporated a discussion of *Wazazi Nipendeni* messages into several episodes. Radio spots were aired on 19 national and regional radio stations with a frequency of 4 to 12 spots per day. Branded materials with *Wazazi Nipendeni* messages, such as T-shirts, bags, and bumper stickers, were distributed at community events in each region. Provider and client print materials such as posters, brochures, SP reminder cards, and pregnancy wheels (a tool used to calculate due date and monitor progress throughout pregnancy) were distributed through the Ministry of Health and Social Welfare and implementing partners to health facilities for reinforcement of messages at the service delivery level. According to partner organization reports, these materials reached approximately 3400 of the nearly 5000 health facilities in Tanzania at the time. All materials also promoted a short-message-service (SMS) platform that allowed women, birth supporters, and others interested in safe motherhood practices to receive free text messages with information and reminders.

The evaluation results reported here assess the relationship between exposure to *Wazazi Nipendeni* in the last month and several behavioral outcomes: 1) women’s individual birth planning (i.e., resolving logistics of delivering in a health facility prior to birth); 2) number of ANC visits; 3) delivery in a health facility; and 4) HIV testing, both oneself and with a male partner. Outcomes related to malaria prevention behaviors are presented elsewhere.

## Methods

A post-hoc evaluation of the *Wazazi Nipendeni* intervention was conducted via facility-based exit interviews with women attending ANC for prenatal or postnatal (up to 6 months) care.

### Study design and participants

Five regions of Tanzania (Mtwara, Lindi, Morogoro, Tabora, and Mwanza) were purposively selected for data collection on the basis of implementing partners’ success in distributing program materials to health facilities. A total of 30 regions make up the country. The approximate populations for the selected regions in 2013 were 1.2 million in Mtwara; 865,000 in Lindi, 2.2 million in Morogoro; 2.3 million in Tabora; and 2.8 million in Mwanza [21]. Health facilities within each district, including hospitals, health centers, and dispensaries, were targeted for data collection. A total of 122 facilities in 18 districts were identified.

### Procedure

Ethical clearance was obtained from the Johns Hopkins Bloomberg School of Public Health Institutional Review Board and the National Institute for Medical Research in Tanzania. Local authorities (regional, district, ward, and village levels) were informed of the study, and the field team contacted health facilities to seek permission to conduct the interviews. Data collection took place from October through November 2013, approximately one year after the *Wazazi Nipendeni* launch.

Eligible women were ANC and postnatal care attendees who were at least 18 years old and pregnant or delivered a baby within the past six months. Women were approached as they exited a health facility, asked for their interest in participating, and assessed for eligibility. Eligible women were read a consent script in Kiswahili and provided oral consent. Data were collected through the use of a structured questionnaire administered via face-to-face interviewing. Data were recorded on mobile tablet devices. Participants received 5000 Tanzanian shillings (~$3.00 USD) for their participation.

### Measures

#### Demographics

Each woman reported her age, education level, employment status, number of live births to date, residence (urban, peri-urban, or rural), and number of household possessions (an indicator of socioeconomic status; range was 0 to 13, with potential possessions including electricity, paraffin lamp, working radio, working television, telephone, mobile phone, iron, refrigerator, plough, generator, toilet, bicycle, and vehicle/motorbike). Although all of this demographic information was collected, we selected covariates in the statistical models based on those variables that have been previously associated with maternal health and HIV testing outcomes in sub-Saharan African contexts. For maternal health outcomes, the number of previous live births, education, age, and residence were included [22]. The covariates for HIV testing during antenatal care were household possessions, education, and age [23, 24].

#### Intervention exposure

Women were asked whether or not they were exposed to *Wazazi Nipendeni* messages in the past month. The sources and frequency of exposure were also assessed.

#### Behavioral outcomes

To evaluate the impact of intervention exposure on outcomes of interest, participants were asked to: 1) describe the elements of individual birth planning and degree of preparedness; 2) report timing of the first ANC visit and frequency of visits throughout pregnancy; 3) report whether or not delivery occurred in a health facility (postnatal women only); and 4) report HIV testing for themselves and their partner(s).

Individual birth planning was assessed by combining 6 items, all rated with Yes (1) or No (0): whether the woman knew her due date, planned for the location of her delivery, planned how to get to the location, whether someone would accompany her to the facility for delivery, whether she prepared necessary items to bring with her, and whether she planned for someone to take care of her family/home while she was away. These 6 items were added together to create one continuous variable, with 6 indicating the greatest amount of birth planning possible using this measure. Women who reported on these items at pre- and post-birth were analyzed together, but birth status was added as an individual control factor.

Women were asked the timing of their first ANC visit, and they also reported how often they received antenatal care during their pregnancy. From the total number of ANC visits reported, one visit was subtracted for women who had given birth to account for their post natal visit. Those women who had recently given birth were asked whether or not they delivered in a health facility. All women were asked about HIV testing for themselves and their partners.

### Analyses

Step-wise linear and logistic regression analyses were conducted to test the effect of program exposure on outcomes. For all analyses, intervention exposure was entered at the first step, followed by the covariates in the second step: age, number of previous live births, education, and region for the maternal health outcomes (birth planning, timing of the first ANC visit, number of ANC visits, and delivery at a health care facility); age, household possessions, and education for the HIV testing outcomes (self and with partner). No covariate was left out in the models for the outcome variables listed at the second step of the analyses in order to fully test the robustness of the campaign exposure effect. The rationale for this approach is that if the intervention was found to be associated with the desired outcomes at the first step (unadjusted), we examined whether the effect still remained after adding covariates that have had known relationships with the outcomes.

## Results

### Demographics

A total of 1708 women completed the interviews: 837 prenatal and 871 postnatal. The majority of participants (66%) were interviewed in rural health facilities, 13% in urban, and 21% in peri-urban. The mean age of the sample was 26.19 years (*SD* = 6.60), and the mean number of household possessions reported was low (*M* = 3.60, *SD* = 1.72). Many women reported having little education, with 1493 (87.4%) reporting primary education or less (of these, 22.1% reported no education at all). The majority of women (62.7%, *n* = 1071) reported being farmers, and a fifth (*n* = 362) reported being housewives. A smaller proportion of women reported owning their own business (*n* = 179, 10.5%), and the remainder reported being a student or employed elsewhere (formally or informally). The employment variable was coded such that 1 = employed in some capacity and 0 = unemployed. 78% of the women reported being employed in some capacity, and the other 22% were either unemployed or housewives. The mean number of live births for the sample was 2.38 children (*SD* = 1.93), and the range varied widely from 0 to 13 births. Demographic data for the sample are presented in Table 1.

**Table 1:** Demographic characteristics of the sample

		No Exposure n (%)	Any source exposure n (%)
Age	18-24	555 (46.5%)	254 (49.8%)
	25-34	459 (38.5%)	196 (38.4%)
	35-44	171 (14.3%)	58 (11.4%)
	45+	8 (0.01%)	2 (0.003%)
Employment	Farmer	796 (66.5%)	275 (53.8%)
	Housewife	260 (21.7%)	102 (20.0%)
	Other	134 (11.2%)	129 (25.2%)
	Unemployed	7 (0.01%)	5 (0.01%)
Number of household possessions	1-2	418 (34.9%)	76 (14.9%)
	3-4	514 (42.9%)	229 (44.8%)
	5-6	230 (19.2%)	151 (29.5%)
	7-10	35 (3.0%)	55 (10.8%)
Residence	Urban	134 (11.2%)	91 (17.8%)
	Peri-Urban	234 (19.5%)	123 (24.1%)
	Rural	829 (69.2%)	297 (58.1%)
Education	None	309 (25.8%)	68 (13.3%)
	Primary	791 (66.1%)	324 (63.4%)
	Secondary	92 (7.7%)	111 (21.7%)
	Post-secondary	5 (0.04%)	8 (0.2%)

### Intervention exposure

Of the 1708 women, 600 (35.1%) reported hearing the *Wazazi Nipendeni* message within one month prior to the interview, with 16.5% reporting daily exposure. Sources of intervention exposure reported are presented in Table 2. Across all intervention sources (except Facebook and blogs, as there were too few participants reporting exposure from those sources), there were statistically significant differences across participants based on their residence, education, and number of household items (all χ^2^ tests with *p*-values ≤ .001), but not age or employment status. Urban residents were more likely to have been exposed to the intervention across all types of exposures compared to peri-urban and rural participants, as were participants with higher levels of education and number of household possessions. Some of these differences can be expected, given that TV and radio access are more likely for those participants who have resources to own such items, and billboards, posters, and other campaign materials may have had greater saturation in condensed areas than in areas with more population dispersion.

**Table 2:** Sources of intervention exposure

Intervention Material	n(%) (total *n* = 600)
radio	500 (83.3%)
brochure	224 (37.3%)
poster	137 (22.8%)
television	123 (20.5%)
billboard	89 (14.8%)
banner	60 (10.0%)
tire cover	45 (7.5%)
sticker	56 (93.0%)
SMS	53 (8.8%)
t-shirt	42 (7.0%)
newspaper	28 (4.7%)
magazine	30 (5.3%)
bag	23 (3.8%)
community event	20 (3.3%)
blog	4 (0.7%)
other	346 (5.7%)

*Note:* More than one source of exposure possible

The total number of intervention sources of exposure was summed to create an exposure index, for which the mean was 0.76 (*SD* = 1.36).^1^ The kurtosis of this variable was very high (over 14), indicating the assumptions of a normal distribution were violated. Many participants reported no exposure to the message (*n* = 1108, or 65.1% of all women in the sample), and smaller and smaller numbers reported one, two, three or more sources. The variable was recoded to have 6 possible values (0-5 sources). This recoding improved the kurtosis value to be acceptable for regression analyses (3.09, *SE* = 0.12). Message exposure had small, but statistically significant relationships (*p*s < 0.01) with region (*r =* −.18), number of live births (*r* = −.06), education (*r* = .28), and number of household possessions (*r* = .29) covariates, but not with age (*p* > 0.05; see Tables 3 and 4).

**Table 3:** Bivariate results of effect of level of campaign exposure on outcomes

		No sources (*n =* 1112)	One Source (*n* = 304)	Two Sources (*n* = 102)	Three Sources (*n* = 55)	Four Sources (*n* = 46)	Five or more Sources (*n =* 88)
Birth Plan	*Mean* (*SD*)						
	Prenatal	3.92 (1.53)	4.15 (1.65)	4.93 (1.17)^a^	4.35 (1.63)	5.07 (0.96)^b^	4.56 (1.54)^b^
	Postnatal	4.67 (1.69)	4.77 (1.64)	4.56 (1.76)	4.95 (1.47)	4.97 (1.63)	5.02 (1.36)
Timing of first ANC visit	*Mean*_*Weeks*_ *(SD)*						
	Prenatal	17.11 (5.70)	17.55 (5.89)	15.23 (5.27)^e^	16.68 (5.43)	20.14 (5.42)^e^	15.49 (5.81)
	Postnatal	16.91 (5.15)	15.58 (5.94)^b^	16.98 (4.82)	17.90 (6.40)	16.65 (5.94)	15.85 (5.09)
Number of ANC visits	*Mean* (*SD*)						
	Prenatal	2.30 (1.25)	2.19 (1.15)	2.61 (1.13)	2.29 (1.03)	2.47 (1.46)	2.38 (1.29)
	Postnatal	2.66 (1.32)	2.93 (1.43)^b^	2.81 (1.29)	3.00 (1.38)	2.87 (1.41)	3.29 (1.82)^b^
Health facility delivery^c^	*% (n for exposure category)*						
	Postnatal	83.6 (574)	85.4 (137)	89.8 (59)	95.2 (21)	96.8 (31)	91.8 (49)
Prenatal HIV testing							
Self	*% (n for exposure category)*	82.4 (534)	81.4 (167)	88.6 (44)	82.4 (34)	80.0 (15)	89.5 (38)
With partner^d^	% (*n for exposure category*)	47.6 (534)	52.7 (167)	52.3 (44)	55.9 (34)	53.3 (15)	57.9 (38)

^a^ Statistically significant difference between number of source exposures versus no source exposure at *p* < .001

^b^ Statistically significant difference between number of source exposures versus no source exposure at *p* ≤ .01

^c^ Statistically significant difference between number of source exposures versus no source exposure at *p* < .01

^d^ Chi square was not statistically significant

^e^ Statistically significant difference between number of source exposures versus no source exposure at *p* ≤ .05

**Table 4:** Statistically significant covariates in the models

	B	*SE* B	β	95% CIs	*p*
Age					
Birth planning	0.04	0.01	0.16	0.02 – 0.06	< .001
Antenatal care 12 weeks or less	0.04	0.01	1.04	1.02 – 1.06	.001
Delivery at a health care facility	0.07	0.02	1.07	1.03 – 1.12	.002
Education					
Birth planning	0.21	0.07	0.08	0.07 – 0.35	.004
Delivery at a health care facility	0.23	0.10	1.25	1.03 – 1.12	.02
Number of prior live births					
Antenatal care 12 weeks or less	−0.20	0.04	0.82	0.75 – 0.89	< .001
Number of ANC visits before delivery	0.06	0.02	0.09	0.01 – 0.11	.01
Delivery at a health care facility	−0.28	0.08	0.76	0.65 – 0.88	< .001

Note: Covariates that were not statistically significant in each model are not presented in this table (e.g., number of household possessions and region)

### Individual birth planning outcomes by exposure

A step-wise linear regression was conducted for the birth planning outcome because the measure was continuous. The mean for the individual birth planning variable was 4.41 (*SD* = 1.64). In the unadjusted model, the number of message sources was a statistically significant predictor at the first step (β = 0.10, *p* < 0.001). After entering the covariates in the second step of the regression model, the model fit improved (*R* ^2^ change = 0.02*, p* < 0.001), and the relationship between the number of message sources and birth planning remained statistically significant, β = 0.09, *p* = 0.001). The more sources from which women reported hearing the *Wazazi Nipendeni* message, the more they planned for the birth of their child.

### Timing of first ANC visit by exposure

A total of 1679 women reported how many weeks pregnant they were before first attending ANC services. The mean number of weeks was 16.85 (*SD* = 5.53, range 0-38 weeks). There were similar proportions of women who attended ANC prior to 12 weeks (*n* = 519; 30.9%) and between 12 to 16 weeks (*n* = 494; 29.4%). The rest of the women attended ANC after 16 weeks into their pregnancies (*n* = 668; 39.8%). The variable was coded to indicate whether the woman received prenatal care within the first trimester (12 weeks or less) or later.

A step-wise logistic regression was conducted, with number of message sources entered as predictors at Step 1 and the covariates entered at Step 2. The Hosmer-Lemeshow test was not significant at both steps (*p*s at 0.29 and 0.28 at Steps 1 and 2, respectively), indicating the models were good fits for the data. Message exposure was not a significant predictor in the first unadjusted model (*p* > 0.05) or in the second adjusted model after the inclusion of the covariates.

### Number of ANC visits by exposure

The mean number of ANC visits for all women in the sample was 2.54 (*SD* = 1.32), with less than 1% of the sample (16 women) not visiting at all, 20.3% (*n* = 346) visiting just once, 34.5% (*n* = 587) visiting twice, 25.1% (*n* = 426) visiting 3 times, 12% (*n* = 204) visiting 4 times, and the remainder (*n* = 129) visiting 5 to 9 times.

An additional linear step-wise regression was conducted with the number of ANC visits entered as the dependent variable. At the first step, the number of message source exposures was a statistically significant predictor in the model, (β = 0.08, *p* = 0.002). Greater numbers of types of exposure to the *Wazazi Nipendeni* message was associated with an increase in ANC visits, and this result remained statistically significant with the inclusion of the covariates at the second step (β = 0.07, *p* = 0.004).

### Delivery in facility outcomes by exposure

Women who had given birth (*n* = 871) reported delivering their babies in many different environments, including dispensaries, hospitals, or their homes. All non-formal health facilities were coded as 0, and all health facilities were coded as 1. Of the 871 women in the postnatal sample, 85.4% (*n* = 744) reported delivering at a health facility, while 14.6% (*n* = 127) reported delivering at home. Over one-third of the women (37%, *n* = 319) reported delivering in a hospital, 26.8% (*n* = 231) reported delivering at a health dispensary, and 22.7% (*n* = 195) at a health center. Of those women who reported a reason for not delivering at a health facility (*n* = 114), 35.1% said they could not get to the facility in time.

A step-wise logistic regression equation was used to test whether message exposure increased the odds of delivery in a health facility. Data for this outcome were available from 868 women who had given birth (3 had missing data). The Hosmer-Lemeshow test was non-significant at both Steps 1 and 2, indicating the data fit the model well. In the first step of the model, the more message sources to which women were exposed, their odds of delivering at a health care facility increased by 14% (OR = 1.14, CI = 1.00 – 1.31, *p* = 0.05). However, when the covariates were entered into the model, the predictive effect of the number of message sources was not statistically significant, *p* > 0.05.

### HIV testing by exposure

A total of 76.6% (*n* = 1308) of women reported getting information about HIV/AIDS during an ANC visit, 88.1% (*n* = 1504) were tested as part of their visit, and of these, 96.3% (*n* = 1449) received their results the same day. Over half of the women (57.6%, *n* = 984) reported their partners were also tested for HIV during one of their antenatal visits, and 59.9% (*n* = 1023) reported knowing their partner’s status.

Another step-wise logistic regression was run to determine whether intervention exposure was associated with women reporting being tested for HIV themselves (independent of their partner). The number of message sources to which women were exposed was not a statistically significant predictor in the model for HIV testing without, and with the covariates added in the second step of the model, (*p*s > 0.05).

### Partner HIV testing

Pre- and postnatal women were analyzed together to see whether the message exposure was associated with partner testing for HIV at an ANC visit. Model fit statistics were acceptable, with a non-significant Hosmer-Lemeshow test at both steps and statistically significant log likelihood model tests. Intervention exposure was a weak but statistically significant predictor of partner testing for HIV at the first step of the model (OR = 1.09, CI = 0.98 – 1.16), with the odds of women being tested for HIV with a partner increasing 9% with each greater number of message sources. After the inclusion of the covariates in the model, the number of message sources did not remain a statistically significant predictor in the model *p* > 0.05.

## Discussion

Over one-third of the survey respondents reported exposure to the *Wazazi Nipendeni* campaign in the month prior to the interview (Table 5). These findings are consistent with nationally representative media monitoring surveys, which have consistently estimated *Wazazi Nipendeni* exposure at 22-46% [25, 26]. The findings suggest radio remains one of the most far-reaching communication channels, with 83.3% of respondents reporting they heard the campaign through radio. Relatively high exposure to materials distributed through health facilities is encouraging, particularly because the evaluation was conducted 10 months after the launch of the campaign and peak distribution activities, when it would not be unreasonable for the health facilities to have exhausted their supply of materials.

**Table 5:** Exposure to *Wazazi Nipendeni* (number of message sources) and its association with study outcomes

Predictor	B	*SE* B	95% CIs	*p*	β	OR
Unadjusted						
Birth planning	0.09	0.02	0.05 – 0.14	<.001	.10	--
First antenatal visit during 1st trimester	0.03	0.03	0.98 – 1.09	.26	--	1.03
Number of antenatal visits before delivery	0.06	0.02	0.02 - 0.09	.002	.08	--
Delivery at a health care facility	0.13	0.07	1.00 – 1.31	.05	--	1.14
HIV testing (self)	0.07	0.06	0.95 – 1.22	.24	--	1.08
HIV testing with partner	0.09	0.03	0.98 – 1.18	.05	--	1.09
Adjusted with covariates						
Birth planning	0.08	0.02	0.03 – 0.12	.001	.09	--
First antenatal visit during 1st trimester	0.02	0.03	0.96 - 1.08	.62	--	1.02
Number of antenatal visits before delivery	0.05	0.02	0.02 - 0.09	.004	.07	--
Delivery at a health care facility	0.08	0.07	0.94 – 1.25	.28	--	1.08
HIV testing (self)	0.07	0.07	0.93 – 1.22	.34	--	1.07
HIV testing with partner	0.05	0.05	0.96 – 1.15	.30	--	1.05

*Note:* Birth planning and number of antenatal visits before delivery were modeled with linear regression, so ORs were not obtained. All covariates described in the analysis section were entered at the second step of each analysis; the outcomes here are only for the exposure effect before (unadjusted) and after (adjusted) their inclusion in the second step of the analysis

Of the key behavioral maternal outcomes analyzed, individual birth planning and greater odds of a higher number of ANC visits were significantly associated with campaign exposure. Early ANC attendance, delivery at a health care facility, and HIV testing (self or with partner) were not predicted by exposure to the intervention. To date, only two other campaigns designed to impact maternal mortality have been evaluated and described in the literature [6] – one demonstrated improvement in birth planning and other associated behaviors [18] and the other improved delivery preparedness [19]. Our findings complement what has been shown in other studies and further demonstrate how an integrated communication campaign can have significant impact on several safe motherhood outcomes. This intervention could serve as an example for programs implemented within health facilities and/or at the national level.

Previous studies have also found that community mobilization, shifts in positive community norms towards early ANC attendance, and partner involvement are important in improving the impact of campaigns focused on maternal mortality prevention [14, 19, 27]. Community mobilization activities combined with the media and facility level interventions outlined here could bring about greater impact. More formative research also needs to be conducted on how to reach male partners with safe motherhood campaign messages and include them in factors leading to positive maternal and child health outcomes, especially in cultures such as those in Tanzania where male involvement is not the norm.

### Limitations

This study was part of a larger process evaluation of the use of facility-level materials designed for health workers. As such, regions and health facilities with strong implementing partner presence were selected because they were more likely to have received the materials. However, this purposeful selection of facilities may have resulted in an overestimation of campaign reach. Surveying women as they exit a health facility also presents a bias in the data, as this suggests respondents are already exhibiting a certain degree of health-seeking behavior and therefore may be more likely to have positive pregnancy behaviors. The study design did not allow for a quasi-experimental examination of the impact on uptake of services because it does not include women who do not use the services. In addition, women attending ANC may have been exposed to the campaign materials at the health facility. We therefore cannot determine causal sequence of events. Finally, it is worth noting that while this study surveyed women only, *Wazazi Nipedeni* targeted both pregnant women and their partners. Further research is needed to explore the effects of the intervention on male partner outcomes as reported by the males themselves.

### Implications for future programming

The results of this evaluation informed the development of the second phase of *Wazazi Nipendeni*, which prioritized increasing intervention exposure. Across many surveys measuring similar interventions in Tanzania, women (and particularly rural women) consistently have lower levels of communication intervention exposure than men, which may be due to differences in access to media (particularly radio and television, which are usually the most commonly reported sources of exposure when considering the entire sample). In the October 2013 monitoring survey for this program, for instance, 54% of urban women were exposed and only 35% of rural women. However, it is worth noting that, since rural women are the largest demographic in Tanzania, when extrapolating to the population, rural women are usually the largest audience in absolute numbers. This is true for *Wazazi Nipendeni*.

The findings also revealed a significant lack of education and employment and low socioeconomic status among surveyed women; making better inroads to underserved populations with low literacy and socioeconomic status is key for improving targeted campaign messages. This is especially true given education remained a significant covariate in the prediction of birth planning and health facility delivery. Community engagement with community health volunteers at the village level needs to be developed and rolled out in coordination with health facilities. Regional and local radio stations could also play a role in disseminating campaign messages to remote locations. In fact, monitoring survey data has shown that program reach in general is higher when regional radio stations are included [25, 26].

One barrier to health facility delivery reported by many women in this study was distance to the facility. As a result of these and other findings, the Tanzanian Ministry of Health and Social Welfare recommends pregnant women move to a location closer to a health facility as their due date draws near [28]. Some government facilities now have associated “waiting houses” that provide a space for pregnant women as they wait for the delivery. Many are situated near or on the same compound as the health center [29]. Including information about this opportunity in campaign messages could further increase the number of facility deliveries.

## Conclusions

Multi-source campaigns that use an integrated approach need to be able to reach pregnant women and their partners with relevant messages for the duration of their pregnancy, not just target specific interventions in isolation, such as SP uptake or ANC attendance. There are many information domains required by pregnant women, including information on proper nutrition, preventive therapies such as SP, and vaccinations such as tetanus toxoid. Approaching women, their partners, and communities with holistic messages linked via multiple communication channels allows for a more streamlined intervention approach, rather than barraging women and their supporters with different messages from a multitude of unconnected campaigns. Harmonizing messages also allows for easier scalability of messages (such as dissemination at the national level), and it allows for scarce resources to be streamlined under an umbrella campaign.

Tanzania is continuing to make progress in reducing maternal and child mortality. Integrated interventions such as *Wazazi Nipendeni* are helping to empower women to take the steps necessary towards a healthy pregnancy, safe delivery, and proper care for newborns. Continued efforts focused on message integration and multiple communication channels will help move Tanzania closer to achieving such benchmarks as the MDGs and CARMMA in the future.

## Abbreviations

ANC: antenatal care
CARMMA: Campaign on Accelerated Reduction of Maternal Mortality in Africa
*CI*: Confidence interval
HIV: Human immunodeficiency virus
IBP: Individual birth plan
ITN: Insecticide-treated net
MDG: Millennium Development Goals
*N*: Sample size
*P*: Probability statistic
PMTCT: Prevention of mother to child transmission of HIV
*SD*: Standard deviation
*SE*: Standard error
SMS: Short message service
SP: Sulfadoxine/pyrimethamine
UNICEF: United Nations Children’s Fund
USAID: United States Agency for International Development
USD: United States dollars
WHO: World Health Organization

## Footnotes

1 A separate index was also created for frequency of exposure to the message; however, frequency reports were very low and not significantly different from exposure indices. All analyses were conducted using both indices, and they did not differ considerably, so only exposure as an index is reported here.
